# Humans modulate arm stiffness to facilitate motor communication during overground physical human-robot interaction

**DOI:** 10.1038/s41598-022-23496-z

**Published:** 2022-11-05

**Authors:** Sambad Regmi, Devin Burns, Yun Seong Song

**Affiliations:** 1 Department of Mechanical and Aerospace Engineering, Missouri University of Science and Technology, Rolla, MO 65401 USA; 2 Department of Psychological Science, Missouri University of Science and Technology, Rolla, MO 65401 USA

**Keywords:** Biomedical engineering, Motor control

## Abstract

Humans can physically interact with other humans adeptly. Some overground interaction tasks, such as guiding a partner across a room, occur without visual and verbal communication, which suggests that the information exchanges occur through sensing movements and forces. To understand the process of motor communication during overground physical interaction, we hypothesized that humans modulate the mechanical properties of their arms for increased awareness and sensitivity to ongoing interaction. For this, we used an overground interactive robot to guide a human partner across one of three randomly chosen paths while occasionally providing force perturbations to measure the arm stiffness. We observed that the arm stiffness was lower at instants when the robot’s upcoming trajectory was unknown compared to instants when it was predicable - the first evidence of arm stiffness modulation for better motor communication during overground physical interaction.

## Introduction

Humans can physically interact with one another without verbal communication^1,2^, such as when assisting an elder to walk or performing a partnered dance. Even without explicitly shared goals, two human partners can physically interact with each other to perform collaborative tasks. Prior work suggests that sophisticated motor communication strategies exist in physically interacting human dyads that can lead to improved performance of the joint task^3–6^, distinction of skill levels^7,8^ or roles^9^, or even motor learning and adaptation to the task^10–13^. Understanding this biomechanical strategy of physical interaction may be the first step in facilitating better physical human-human interaction (pHHI) and ultimately in developing safe and effective physical human-robot interaction (pHRI)^14^.

A promising focal point for observing such communication during pHHI or pHRI may be human arm impedance^15^. During physical interaction, human arms are used as a medium for two-way motor communication^6,16,17^. Since interactions through arms can be characterized by their movements and the interaction forces, the causal relationship between these two physical quantities, which is mechanical impedance, may reveal human motor control strategies. Indeed, previous works investigated arm stiffness (the spring-like component of impedance) to study motor control in humans for stability tasks^18–20^ or in seated reaching tasks^21–25^.

Moreover, it was recently suggested that the arm stiffness may affect perception of small forces and/or movements, which is a crucial factor for effective motor communication^26^. This implies that during practical overground interaction tasks, such as walking assistance, humans may modulate their arm stiffness for increased sensitivity depending on the ongoing interaction dynamics, particularly in the absence of verbal communication or visual feedback. This further motivates the investigation of the arm stiffness during pHRI or pHHI to uncover the underlying motor communication strategy.

To this end, this work presents the first measurement and analysis of human arm stiffness during overground physical interaction between a robot leader and a human follower. The recently developed overground interactive robot, Ophrie^27,28^, was used in an overground pHRI experiment. The task was designed to simulate a guided walking task such that the human subjects were compelled to focus on the movement of the robot to understand its movement intention. We applied perturbations to measure arm stiffness at two importantly different time points during the trials: either at the point where three potential robot trajectories diverge such that the subjects are least certain of their path, or near the end of the trial when the subjects are most certain that the robot always continues straight forward. By comparing the human arm stiffness in these two conditions that require different levels sensitivity of motor communication, we present the first evidence of human arm stiffness modulation for effective overground pHRI.

## Results

**Figure 1:**
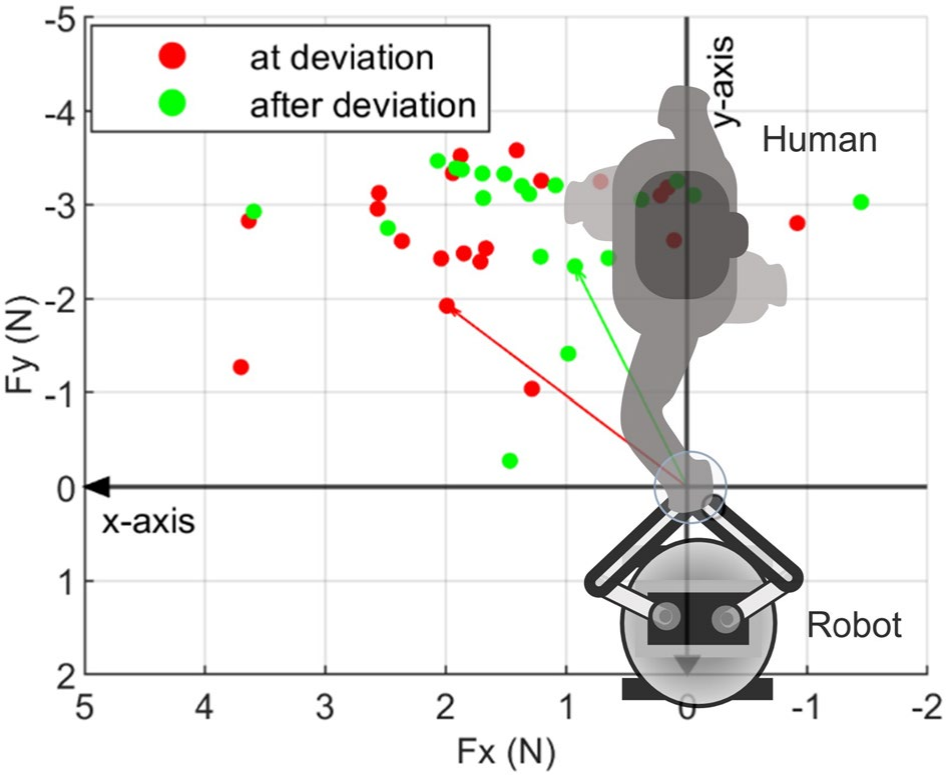
The interaction forces due to background stiffness for all trials performed by a representative subject. The dots represent the interaction forces presented to the humans just before the perturbation onset during the ‘at deviation’ condition (red) or the ‘after deviation’ condition (green).

**Figure 2:**
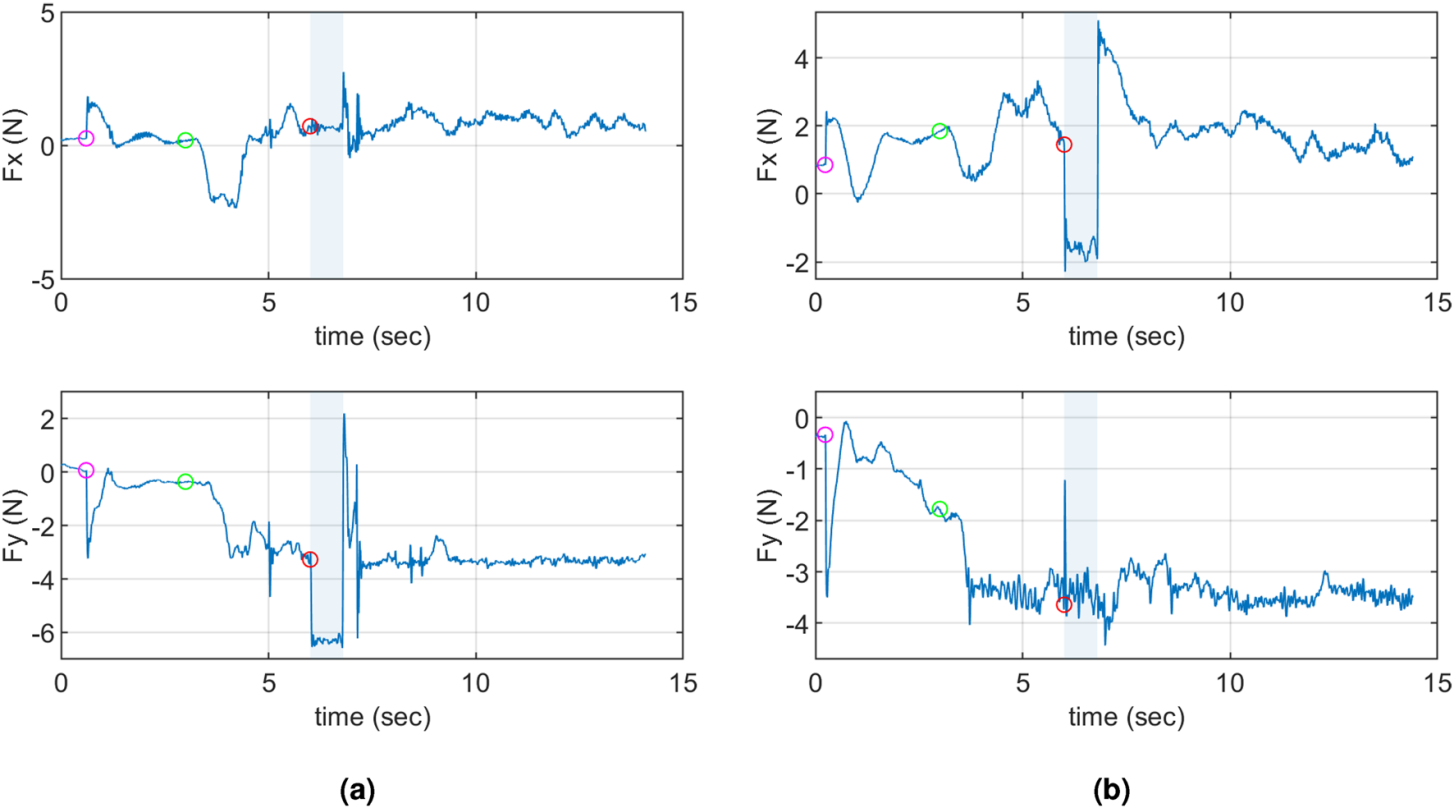
The interaction forces during the entire duration of two representative trials of subject 1. Pink marker: the instant when the position-based background stiffness controller was activated. Green marker: the instant when the robot started moving and the subject began walking with the robot. Red marker: the instant when the force perturbation was initiated; the blue shaded region shows the 800 ms duration of the perturbation. The earlier portion of this 800 ms window is used for arm stiffness estimation. (**a**) is the representative lateral (-*y*) perturbation and (**b**) is the representative forward (-*x*) perturbation.

**Figure 3:**
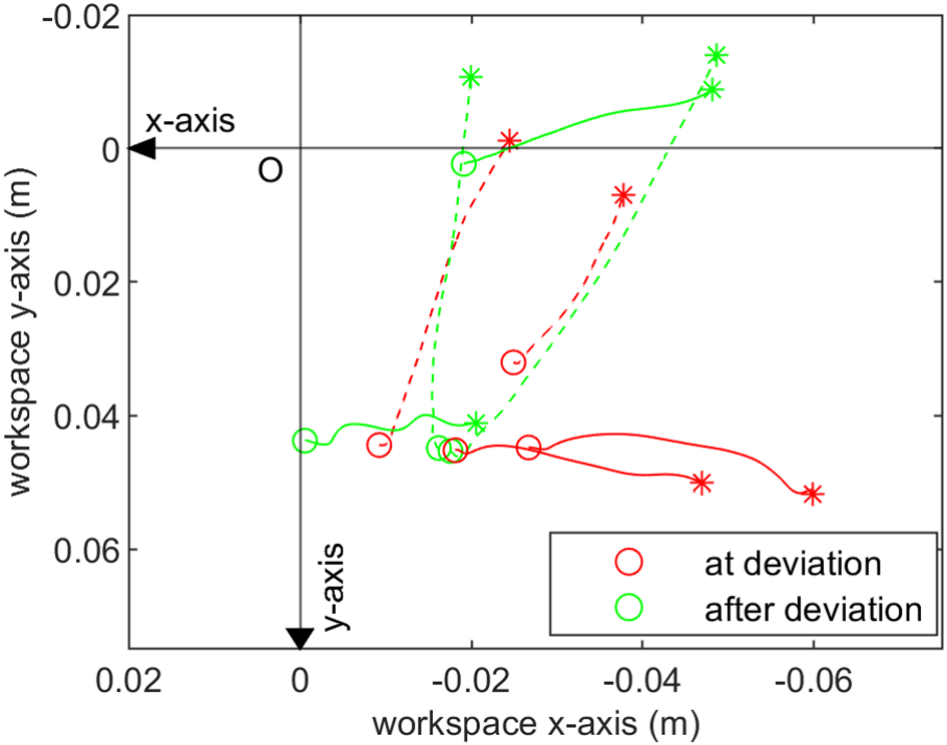
The hand movement trajectories from block 1 of subject 1. The hand is at the circle (o) at the perturbation onset and is displaced to the asterisk (*) after 300 ms. Red and green colors represent the perturbation applied during ‘at deviation’ and ‘after deviation’ trials, respectively. The solid and dotted lines represent the hand movement due to the forward (-*x*) and lateral (-*y*) perturbations, respectively.

Subjects walked with an interactive robot while closing their eyes and holding the handle on the robot arm. The robot moved on one of three trajectories that involved straight and deviated segments. When asked to guess which trajectory the robot moved on, the subjects were able to respond correctly in 386 out of 400 total trials, implying that the subjects were paying close attention to the movement of the robot throughout the experiment. The average interaction force among all the subjects and all trials due to background stiffness right before the perturbation onset was observed to be a pushing force of 2.215±0.976N. That is, on average, the subjects were pushing the robot towards their front-right direction and the resulting reaction force from the robot was in the opposite direction. This can be seen in Fig. 1 where +y is towards the robot and -x is the forward movement direction. When perturbation was applied, the perturbation forces of 3 N were imposed on top of the current interaction force at that moment, briefly increasing the interaction force magnitude in either -x or in -y direction (at 6 s, Fig. 2). As a result, the subjects’ hands were displaced approximately towards -x or -y direction as can be seen in Fig. 3.

**Figure 4:**
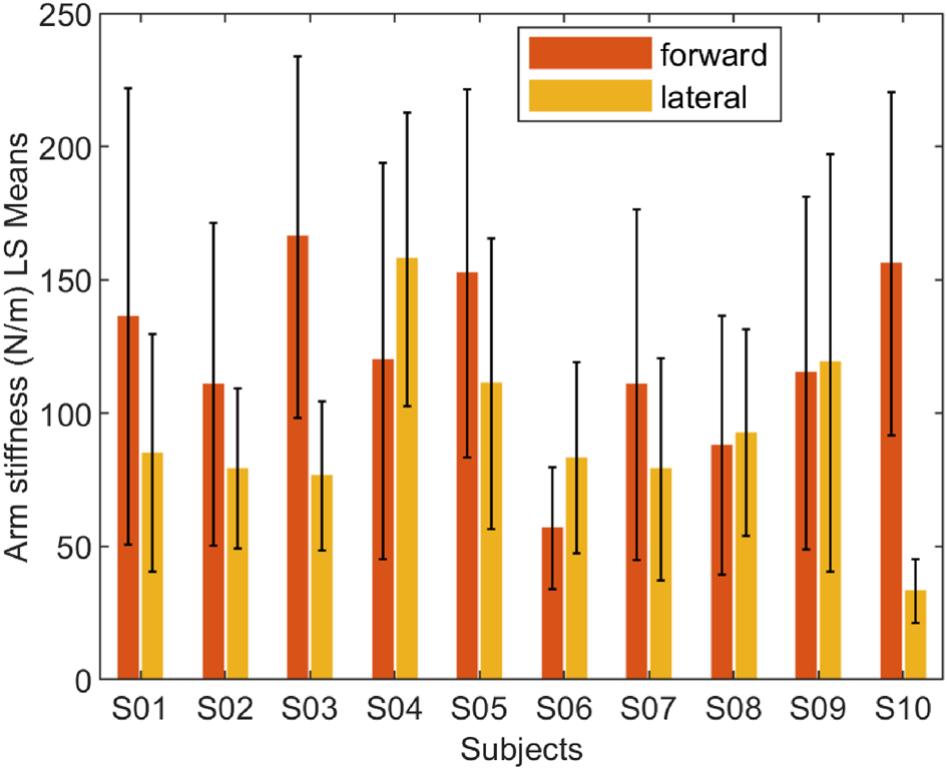
The mean and the standard deviation of individual subject’s arm stiffness based on perturbation direction in both at- and after-deviation conditions.

**Figure 5:**
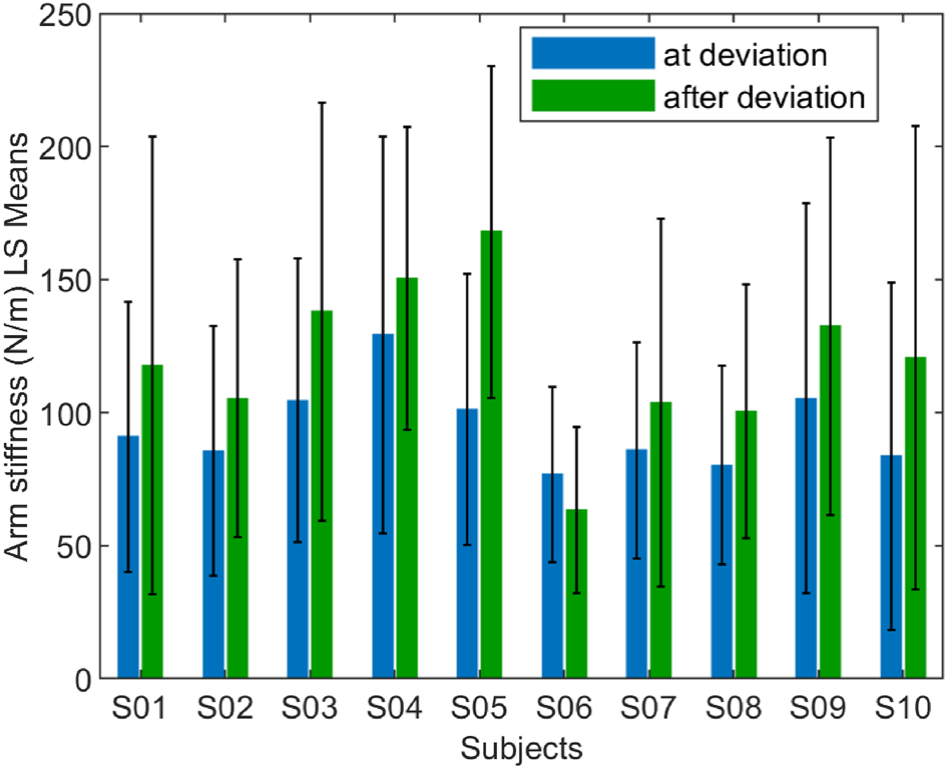
The mean and the standard deviation of individual subject’s arm stiffness based on the condition in both forward and lateral perturbations.

The interaction dynamics were estimated using linear regression, as discussed in the ‘data acquisition and analysis’ section. Out of 400 trials, 28 trials were excluded from analysis due to the estimated stiffness being outliers. The average stiffness across all subjects and conditions is 106.819±63.741 N/m. The stiffness values for different conditions and perturbation direction can be found in the Table 1, which is consistent with the literature on unconstrained voluntary arm movements^22^. The mean arm stiffness and the standard deviation for the individual subjects based on the perturbation direction (forward or lateral) is shown in Fig. 4, and based on the condition (‘at deviation’ or ‘after deviation’) is shown in Fig. 5.

The acceptability of the linear regression result was evidenced by the low positive estimates of endpoint inertia (0.207±0.275 kg) and damping (9.114±8.321 Ns/m) parameters, which were expected of relaxed human arms and with the low intrinsic mechanical impedance of the robot arm. The mean equilibrium position of the hand was estimated to be at 0.027±0.045 m (averaged over all trials and all subjects) away from the workspace center towards the robot. To further address the reliability of the interaction dynamics estimation, the interaction forces were estimated using the estimated inertia, damping, stiffness, and the measured hand trajectory. This was then compared against the actual interaction force. The mean and standard deviation of the *R*^2^ values between the estimated and measured interaction forces of all 40 trials were 0.568±0.108 (subject 1), 0.567±0.087 (subject 2), 0.533±0.078 (subject 3), 0.578±0.084 (subject 4), 0.528±0.047 (subject 5), 0.559±0.062 (subject 6), 0.539±0.072 (subject 7), 0.516±0.040 (subject 8), 0.520±0.071 (subject 9), and 0.582±0.175 (subject 10).

**Table 1:** Average stiffness across all subjects and conditions.

Subject ID	Stiffness(N/m)			
	Forward - after deviation	Forward - at deviation	Lateral - after deviation	Lateral - at deviation
Subject 1	155.344±95.954	112.086±68.878 (2)	79.877±56.844	90.146±30.395
Subject 2	126.042±60.257 (1)	97.043±60.672	84.684±34.610 (1)	74.228±26.283
Subject 3	198.327±65.804	133.836±55.068	77.712±24.215	75.299±32.362
Subject 4	161.686±70.574 (2)	85.858±60.991	139.174±60.969 (2)	172.567±61.956
Subject 5	180.310±71.092	124.451±57.434	152.453±49.584 (2)	77.800±30.854
Subject 6	52.734±19.204	61.268±26.444	74.096±38.194	92.290±32.350
Subject 7	137.792±83.859	83.582±21.155	69.715±21.693	88.058±54.952
Subject 8	114.512±55.413	61.315±17.850	86.253±35.528	98.980±42.689
Subject 9	185.360±44.275 (5)	76.039±36.319 (1)	106.118±68.003	131.581±89.025
Subject 10	177.946±62.919 (1)	127.900±58.163 (3)	34.457±8.807 (4)	31.909±15.304 (4)

The number of discarded trials are shown in the parentheses.

**Figure 6:**
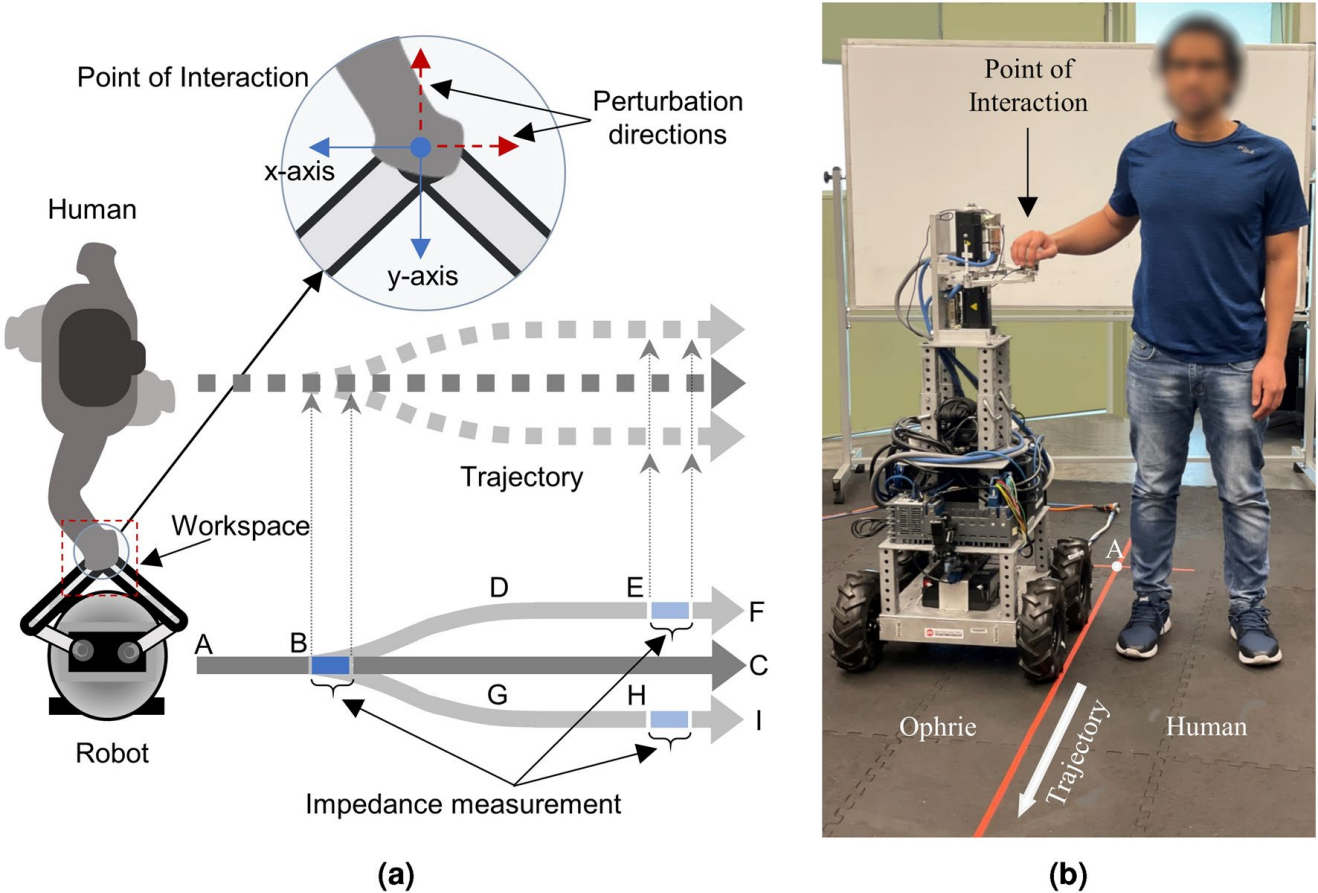
(**a**) Schematics of the overground pHRI experiment setup (top view). The horizontal length of AB, BC, BD/BG, DE/GH, and EF/HI are 1.5 m, 3.95 m, 1.25 m, 1.85 m, and 0.85 m, respectively. D and G (and therefore F and I) were 0.5 m apart. (**b**) A research personnel holding the robot handle to demonstrate the pose at the beginning of a trial.

The skewness and the excess kurtosis of the estimated stiffness values were 1.275 and 1.472, which indicated that the distribution cannot be considered substantially non-normal^29^. Therefore, parametric tests can be utilized for analysis. The stiffness data between the two curved trajectories (curved towards the subject or away from the subject, Fig. 6) were compared with a paired t-test and were shown to be not significantly different (*p*>0.780; least square means of 120.099 N/m and 127.174 N/m). Hence, stiffness measurements from all curved trajectory trials were grouped and labeled as a single ‘after deviation’ condition. All stiffness measurements from the straight trajectory trials were labeled as ‘at deviation’ condition.

**Figure 7:**
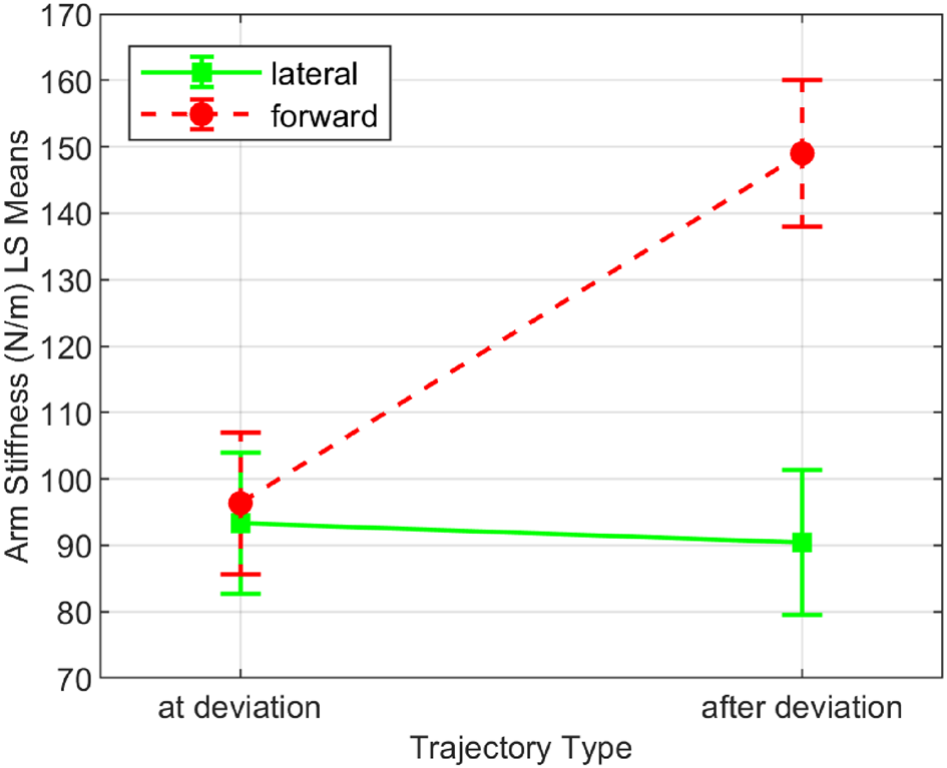
The effects of perturbation direction on the arm stiffness (least square means, 95% confidence interval) based on the condition, in all subjects.

We performed a three-way ANOVA with the following factors: subject—10 levels, conditions—2 levels (‘at deviation’ and ‘after deviation’), and perturbation direction—2 levels (‘lateral’ and ‘forward’). The omnibus test (Table 2) showed that the mean of at least one combination in the ANOVA model is significantly different from others (p<0.001). Subsequent interaction effect analysis (Table 3) revealed that the three-way interaction of the included factors is not significant (*p*>0.090). Likewise, there is no significant interaction between the subjects and the conditions (*p*>0.179, see Fig. 5). In contrast, a strong interaction between the subjects and the perturbation direction was observed to affect the arm stiffness (*p*<0.0001), which implies that the trend in variation of the arm stiffness in lateral to forward direction is not consistent among subjects (see Fig. 4). Likewise, a significant interaction between the experiment design parameters (perturbation direction and experiment conditions) is observed (*p*<0.0001). This suggests that the arm stiffness in different directions varies differently based on when it was measured—that is, before or after the deviation in Fig. 7.

The main effect of the conditions on stiffness estimates was significant. The stiffness of the arm was significantly higher in the ‘after deviation’ condition than in the ‘at deviation’ condition (118.604±70.247 N/m and 95.530±54.661 N/m, respectively, *p*<0.0001). That is, the arm stiffness was lower when the robot trajectories may or may not make a curve (B in Fig. 6) compared to when the robot trajectories were always straight (E or H in Fig. 6). The main effect of subject was also significant (*p*<0.001) showing strong inter-subject variability. The main effect of the perturbation direction was also significant (*p*<0.0001), consistent with the well-known elliptical shape of 2-D stiffness reported in the literature^22,30^.

**Table 2:** Analysis of Variance.

Source	DF	Sum of squares	Mean square	F ratio
Model	39	605320.2	15521.0	5.7126
Error	332	902033.6	2717.0	**Prob**>**F**
C. Total	371	1507353.8		<**0**.**0001**$^{*}$

Significance values are in bold.

**Table 3:** The main and interaction effects of the factors.

Source	Nparm	DF	Sum of squares	F Ratio	Prob>F
Subject	9	9	165466.11	6.7668	<**.0001**$^{*}$
Condition	1	1	56182.72	20.6785	<**.0001**$^{*}$
Perturbation direction	1	1	85846.42	31.5964	<**.0001**$^{*}$
Subject$*$condition	9	9	34697.11	1.4189	0.1785
Subject$*$perturbation direction	9	9	174534.85	7.1376	<**.0001**$^{*}$
Condition$*$perturbation direction	1	1	69678.12	25.6455	<**.0001**$^{*}$
Subject$*$condition$*$perturbation direction	9	9	41356.29	1.6913	0.0899

Significance values are in bold.

## Discussion

The movement trajectories of the robot were specially designed to capture the contrast in overground arm stiffness between (a) when the subjects would be compelled to pay attention to the haptic cues from the robot and (b) when they are not compelled to. The former is implemented as the ‘at deviation’ trials where the subjects were perturbed at the exact location at which the robot turns in 50% of the trials (B in Fig. 6). In this condition, we expected the subjects to actively “listen” to the robot through their hand because they were asked to guess the trajectory of the robot and follow its lead. In addition, the subjects had their eyes closed and were not told whether the robot would turn or not for any specific trial, and hence they had to rely on the information coming through their arm and hand which is holding the robot’s handle. On the other hand, once the robot has passed the first half of the trajectory, the remainder of the path was always straight in the forward direction. In these ‘after deviation’ conditions, the arm stiffness was measured long after the robot completed the turn, when the subjects did not have any compelling reason to listen to the haptic information from the robot because it’s motion was predictable. The subjects reported the trajectory correctly 96.5% of the time (386 out of 400 trials) which indicates that they were actively sensing the robot movement, at least up to the trajectory deviation.

In general, the subjects pushed the robot towards their front-right (-*x* and +*y* in Fig. 1). This may be because the eyes-closed subjects expected the robot which is leading them to be slightly ahead of them to the right, and that they preferred to keep a safe distance from the robot by pushing it away. While maintaining this push towards the front-right, the subjects may have sensed the forward movement of the robot by sensing a decrease in the intensity of the push in the forward direction.

We observed that the arm stiffness was significantly different in ‘at deviation’ condition trials compared to ‘after deviation’ condition trials (*p*<0.0001). This indicates that humans modulate their arm stiffness during overground physical interaction tasks. The modulation was related to whether they were compelled to listen to haptic information (i.e. ‘at deviation’ condition) or not (i.e. ‘after deviation’ condition). Note that the arm stiffness modulation was completely voluntary - the subjects were free to choose how stiff or compliant their arm could be during the entire experiment. The results imply that the subjects may have lowered their arm stiffness in the forward direction due to the need for more sensitivity in haptic communication ‘at deviation’. When less sensitivity was required ‘after deviation’, their forward arm stiffness was higher. In the lateral direction, this modulation did not occur, which may imply that the need for communication was less in that direction.

Our result is consistent with the hypothesis in our prior study^26^ that low arm stiffness may facilitate motor communication. According to^26^, lower arm stiffness may allow larger movements to occur from small interaction forces, which will then be better sensed by the proprioceptors in the musculoskeletal system. Our results reinforce this observation. The key distinction is that, while in^26^ the stiffening of the arm influenced the sensitivity to small interaction forces, in this work, the need for better sensitivity caused the subjects to modulate the stiffness of their arms. Also, in^26^ the arm was heavily constrained such that the arm configuration was predetermined and fixed with respect to the position of the robot. In contrast, in this work, the arm configuration and the hand position with respect to the robot were allowed to deviate. This allowed the subjects to modulate their arm movement as desired in a more naturalistic way. Hence the state of the arm (such as how stiff it is) was decided freely by the subject as needs arose. This study implies that the said ‘need’ could be the need for higher sensitivity during times when the robot’s movement was more unpredictable.

The modulation of the stiffness in the forward direction was significant, whereas in the lateral direction, the modulation was not significant (Fig. 7). This may imply that the subjects were more concerned about sensing the forward movement rather than the lateral movement of the robot. Indeed, due to the non-holonomic constraint, the wheeled base of our robot cannot generate strictly sideways movement. Hence the subjects may not be as compelled to modulate the arm stiffness in the lateral direction as much as in the forward direction.

Another possibility is that the modulation of stiffness may have been a result of the change in the arm posture^30^. The shape and size of the 2-D stiffness ellipse depends on the posture^15,22^, where the ellipse eccentricity is higher as the hand gets further away from the chest. In our study, the forward stiffness is higher than the lateral stiffness in the ‘after deviation’ condition, indicating high eccentricity and suggesting that the hand may have been further away from the chest than it was during the ‘at deviation’ condition. The modulation of stiffness with posture (instead of muscle activity) is more likely given the low stiffness values that are comparable to the stiffness of the relaxed arms in^22^. Future experiments with arm kinematics and electromyography may address this hypothesis.

The main effect of the subjects was significant, which was expected because every individual is different and might respond to similar perturbation differently. It is not uncommon to have higher inter-subject variability in experiments involving humans, especially with regards to bio-physiological variables such as arm stiffness. More variance could have been added due to the nature of the experiment having high degrees of freedom as an overground experiment (as opposed to constrained, seated settings). Factors such as the subjects’ walking habits (posture), height, or arm muscle cross-section all would contribute to this variability.

While the robot’s velocity setpoint did not differ by trials in this experiment, future usage of Ophrie may include applications with varying velocity profiles including the maximum speed. Our observations suggest that the human user’s arm stiffness would be lower when predictability is lower with the robot partner. The robot’s speed, by itself, does not increase or decrease predictability and therefore may not affect the arm stiffness of the user. However, the rate at which the robot reaches its setpoint speed (fast or slow) may motivate the human user to change their arm stiffness values, since higher acceleration may be perceived as lower predictability. For example, an overall slower robot with frequent periods of high acceleration and deceleration may be perceived as less predictable than a faster robot with a slow rate of change of speed.

The arm stiffness modulation observed in this study has further implications in the design and control of robots for overground pHRI^31^. It may be argued that the robot partner would feel more human-like to the user if the robot arm’s mechanical impedance was similar to that of a typical human. In this view, the low impedance of the robot arm may provide an impression of attentiveness to the user’s intent. The robot may also selectively lower the stiffness in specific directions to communicate its movement intent or role-taking to the user^12^.

## Methods

### Participants

A total of 10 healthy young adults (1 female, 1 left-handed, 27.6±2.41 years) without any self-reported neurological disorder took part in the study. Subjects signed a written informed consent form before participating in the experiment. The research protocol was approved by the Institutional Review Board of the University of Missouri System, and was performed in accordance with relevant guidelines and regulations.

### Experiment protocol

A guided walking experiment was designed wherein the human participants were asked to walk alongside the robot Ophrie—that acted as a leader—while holding the robot’s handle. Ophrie was specially designed for the overground pHRI experiments. Its manipulator possesses unique characteristics that are critical to overground physical interaction experiments involving humans, such as low inherent endpoint impedance, the ability to apply and measure small force perturbations (<10 N), and the capability of endpoint movement speed of 0.845m/s assuming cyclic motion of 2 Hz within the workspace^27,28^.

In the experiment, subjects were asked to walk following the lead of the robot. During each trial, the human participants were asked to stand upright next to Ophrie and held its handle using their right hand as shown in Fig. 6b. The robot’s height was adjusted beforehand such that the height of the interaction handle was aligned to the subject’s elbow level. The subjects were informed that the position-based background stiffness controller (explained below) would be activated after they are ready, and hence they should relax their arm to let the robotic arm position the handle to the center of its workspace—a 0.15*x*0.15 m^2^ 2-dimensional space parallel to the ground plane (see Fig. 6a). The subjects were informed that, after the experimenter issues a verbal cue, the robot would start to move, and they had to follow the robot with their eyes-closed throughout the entire trial.

The background stiffness controller provided an interaction force to the human user such that the subjects’ hands were maintained near the center of the workspace, such that

1 $$\begin{aligned} \begin{bmatrix} F_x \\ F_y \end{bmatrix} = -K \begin{bmatrix} 1 &{} 0 \\ 0 &{} 1 \end{bmatrix} \begin{bmatrix} x \\ y \end{bmatrix} \end{aligned}$$

where (*x*, *y*) is the location of the end point with respect to the center of the workspace (0, 0), *K* is the scalar level of background stiffness, and ($F_x$, $F_y$) are the forces generated by the robot.

The robot was programmed to go on a straight trajectory for the initial 1.5 m, then the trajectory would deviate either to the left or right, or remain straight in the forward direction (Fig. 6a). The deviation (BD or BG in Fig. 6a) took 3.8 s. After that, the trajectories were always straight. The paths for these three different trajectories were marked on the floor using colored tapes to ensure that, prior to the beginning of the experiment, the subjects clearly understood (a) where the robot might deviate from straight trajectory and (b) the experiment design that “the latter half of the trajectory will always be straight”. Given their eyes were closed, the subjects were expected to listen to haptic information felt from holding the interaction handle to sense the movement of the robot and the deviation in the trajectory when the robot turned. After the robot stopped, the subjects were asked to tell in which direction the robot deviated based on their judgment before opening their eyes.

The top view sketch of the entire experimental setup and the ideal starting pose for each trial is shown in Fig. 6. Throughout the entire trial, the robot was programmed to ramp up to and maintain a constant linear velocity of 0.5 m/s. When turning, the angular velocity ramped up to 0.45 rad/s and then ramped down to zero. In straight trajectory trials (ABC in Fig. 6a, 50% of the total number of trials), the robotic arm applied a force perturbation of 3 N (lasting 0.8 s) when it reached B. In trials with deviated trajectories (the other 50% of the trials equally divided between ABDEF or ABGHI in Fig. 6a), the perturbation was applied at either E or H. The perturbation was applied either towards the -*x* or the -*y* direction. Subjects were aware that the robot might turn and that it will also apply a slight push at some point on the trajectory, but they were unaware of the direction of the turn or the direction of perturbation for any specific trial. The presence of a background stiffness controller helped the subjects to maintain their hand inside the workspace; the stiffness controller was briefly turned off during the perturbation, so that the estimated stiffness within the 0.8 period would be solely due to the arm stiffness. Each trial took approximately 20 s.

In this work, we applied perturbations at one of two different time points during the trials to measure stiffness at different levels of participant attention to the robot movement. The first time point is ‘at deviation’, which happens at point B (Fig. 6a), where the three potential paths deviate. In all of the trials that used this early perturbation, participants continued on the straight path BC after perturbation, since the perturbation would interfere with information required to signal a turn. Since subjects did not know ahead of the trial whether the trajectory would be straight or curved, they would be uncertain to which trajectory the robot would follow after point B - to go straight, or to curved to the left or right, and thus paying close attention to the robot’s movements. The other time point is ‘after deviation’, which happens at the end of the trial at point E or H (Fig. 6a), when the robot always continues straight for the remainder of the trial. These perturbations always occurred when the robots took one of the curved paths, since the straight path trials used the earlier perturbation. Since subjects already knew this, there was no uncertainty about the robot’s trajectory at point E or H. The label ‘after deviation’ came from the fact that the perturbation occurred many seconds after the robot has chosen its trajectory. For any given trial, there was exactly one perturbation, occurring either at point B (‘at deviation’ condition) or at E/H (‘after deviation’ condition).

The arm stiffness measurements were made in two orthogonal directions (-*x* and -*y*) to capture the directional differences in the arm stiffness. The choice between +*x* and -*x* or between +*y* and -*y* could be made arbitrarily since the model used in this study and in the literature^15^ assumes linearity. It is well known that the 2D-constrained arm presents a 2D stiffness ellipse^15^. Since arm stiffness is affected by the arm configuration^15,30^, the measurement of stiffness ellipse requires that perturbation is provided at the same arm configuration across multiple trials. This was possible for seated experiments such as^22^ where the subject’s trunk location was fixed with respect to the robot. However, this is not possible for overground pHRI experiments where the trunk location with respect to the robot must be allowed to vary (ex. natural lateral sway due to walking). Thus the two directional arm stiffness were measured and reported as individual scalar values. The number of directions was limited to two to avoid excessively long experiments that could result from having three or more perturbation directions. The varying arm configuration in overground pHRI experiments was also why force perturbation was chosen over position perturbation in this study, since the latter requires a knowledge of the expected position or trajectory of the arm movement at the time of perturbation^32^.

In summary, there were six different trial types depending on the direction of trajectory deviation (towards F, C, or I, 3 levels) and the direction of force perturbation (2 levels). The experiment was designed as a Randomized Complete Block (RCB) design. Each block consisted of eight randomly distributed trials where there were two ABDEF, two ABGHI, and four ABC trajectories. The ABC trajectory constituted the ‘at deviation’ condition and the two deviated trajectories constituted the ‘after deviation’ condition. There were the same number of trials for straight versus deviated trajectories such that the same number of measurements could be made for ‘at deviation’ and ‘after deviation’ conditions. Each of these groups had an equal number of forward and lateral perturbations. There were 5 blocks totaling the number of trials per subject to 40. Each experiment on one subject took approximately 2.5 hours.

### Data acquisition and analysis

The interaction dynamics is the function of arm movement and its derivative. A widely used simplified model was adopted in this study where we assume the interaction dynamics in task space as passive, linear, and time-invariant second-order dynamics^33–36^.

2 $$\begin{aligned} f - f_0 = m~(\ddot{x} - \ddot{x}_0) + b~(\dot{x} - \dot{x}_0) + k~(x - x_0) \end{aligned}$$

where *f* is the interaction force measured after the onset of force perturbation, *x*, $\dot{x}$, and $\ddot{x}$ are the position, velocity and acceleration of the hand after the perturbation, respectively, and *m*, *b*, and *k* are the inertia, damping, and stiffness from the interaction dynamics, respectively. $x_0$, $\dot{x}_0$, and $\ddot{x}_0$ are the mean hand position, velocity, and acceleration of the interaction point just before the perturbation, and likewise, $f_0$ is the interaction force just before the perturbation, averaged over the 0.1 second interval prior to the perturbation onset to reduce the effect of high-frequency noise in force measurements.

**Figure 8:**
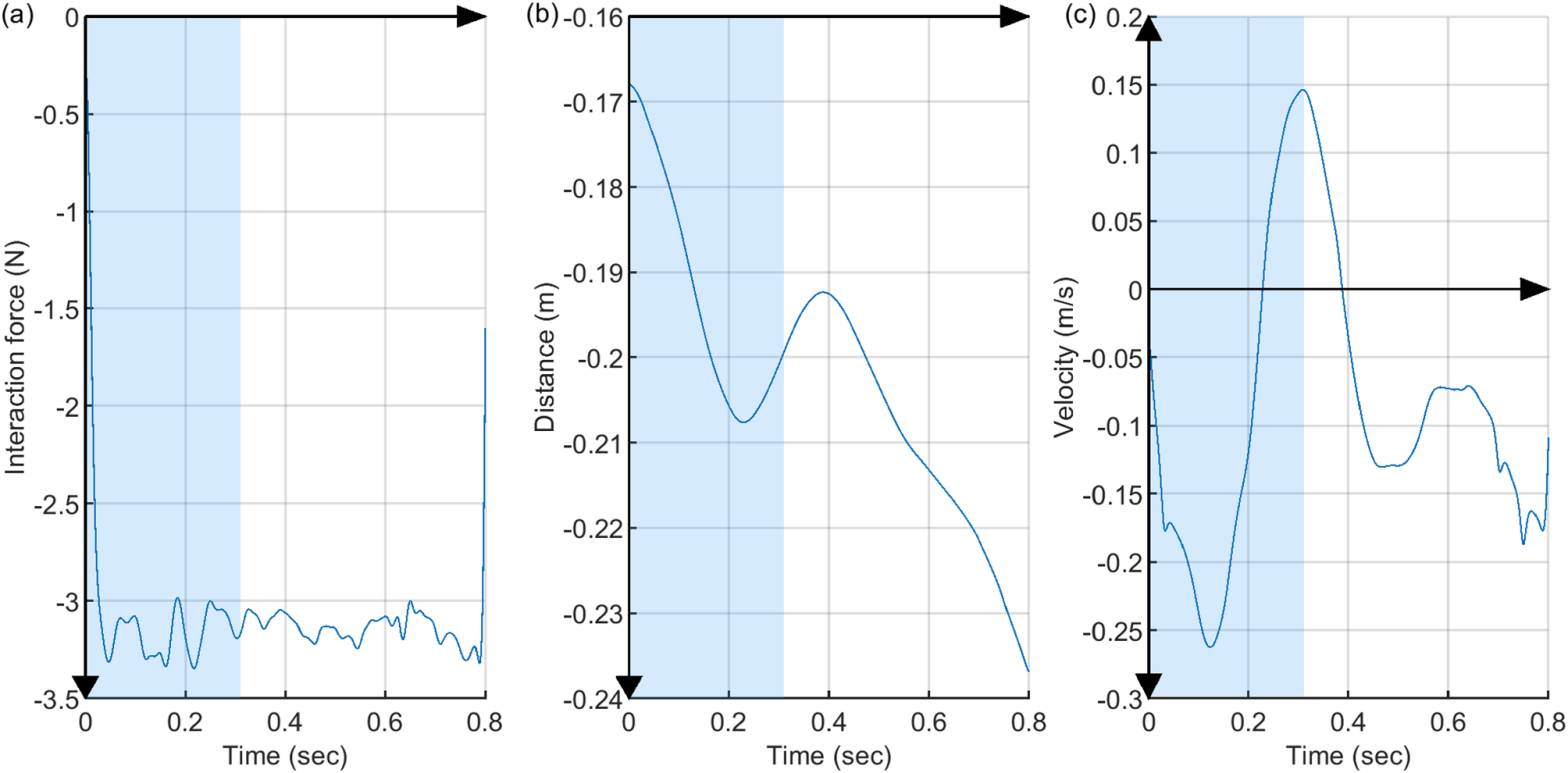
Measurements during the 800 ms perturbation of a representative trial of a subject. The shaded area represents the time stamp from the perturbation onset to the time of second peak in the velocity plot. The data from this shaded region was used to estimate the arm stiffness. (**a**) The interaction force between the robot arm and the human subject. (**b**) The position of the hand with respect to the origin of the workspace in y-direction. (**c**) The velocity of the hand in y-direction.

The hand position with respect to the robot can be calculated using the motors’ encoder positions and forward kinematics. The motor shaft position was measured with the single-turn absolute Biss sine encoder (2048 LPR) embedded in the servo motors (AKM32E-ANCNAA00: Kollmorgen Corp, VA, USA). The kinematic details of the robot arm can be found in^27^. The interaction forces were measured using the mini45 force/torque sensor (ATI Industrial Automation, NC, USA), which is lodged between the endpoint of the robotic arm and the interaction handle. All data were recorded with a sampling rate of 1 KHz. After recording, the hand position and force data were filtered in Matlab (MathWorks Inc., MA, USA) using a Butterworth low-pass zero-lag filter with 40 Hz cutoff frequency.

From the measured hand position data during perturbation, we used the earlier portion of the arm movement response that shows the characteristics of a passive second-order system. First, the hand velocity versus time graph was plotted in Matlab and the time instant of the second peak (local maximum) was manually identified, since the general trend of the arm movement up to this instant were similar across subjects and also resembled the second-order response. Then, the time interval from the perturbation onset to the time of second peak was selected as the period of data for analysis—which was on average 438.5±113.7 milliseconds. The perturbation force, hand position, and hand velocity during the 800 ms perturbation for a representative subject can be seen in Fig. 8. Multi-variable linear regression (Matlab fitlm function) was applied to this data to estimate *m*, *b*, and *k* in Equation 2. The stiffness (*k*) is the main outcome of interest, whereas the inertia (*m*) and the damping (*b*) were only used to check the validity of the estimation.

Each trial from the experiment fetched one scalar stiffness value in either forward or lateral direction. It can be considered as the arm’s resistance to an external perturbation towards the axis of the perturbation. Out of 400 trials performed by 10 different subjects, 17 were discarded because the stiffness estimates were negative. From the remaining 383 trials, 11 outliers—defined as values more than 3 times the interquantile range past the 25^th^ or 75^th^ quantiles—were screened out using JMP statistical software (SAS Institute Inc., NC, USA). ANOVA was performed using JMP on the remaining 372 stiffness values. A significance level of *p*=0.05 was used.

## Data Availability

The datasets generated and/or analysed during the current study are available in the Harvard Dataverse repository, https://doi.org/10.7910/DVN/XEKVUF.
